# Associations of polygenic risk scores for major depression and depression severity: an investigation of 105 623 individuals with 16 years follow-up

**DOI:** 10.1038/s41380-025-03243-2

**Published:** 2025-09-17

**Authors:** Marit Haram, Andreas Jangmo, Piotr Jaholkowski, Joëlle Pasman, Joeri Meijsen, John R. Shorter, Elizabeth C. Corfield, Oleksandr Frei, Ted Reichborn-Kjennerud, Alfonso Buil, Yi Lu, Thomas Werge, Patrick Sullivan, Ole A. Andreassen, Martin Tesli

**Affiliations:** 1 Institute of Clinical Medicine, University of Oslo, Oslo, Norway; 2 Division of Mental Health and Addiction, Oslo University Hospital, Oslo, Norway; 3 Department of Mental Health, Norwegian Institute of Public Health, Oslo, Norway; 4 Department of Medical Epidemiology and Biostatistics, Karolinska Institute, Stockholm, Sweden; 5 Amsterdam UMC location University of Amsterdam, Department of Psychiatry, Genetic Epidemiology, Amsterdam, The Netherlands; 6 Institute of Biological Psychiatry, Mental Health Center Sct. Hans, Mental Health Services Copenhagen, Roskilde, Denmark; 7 The Lundbeck Foundation Initiative for Integrative Psychiatric Research (iPSYCH), Copenhagen, Denmark; 8 Center for Eating and feeding Disorders Research, Mental Health Center Ballerup, Copenhagen University Hospital, Mental Health Services Copenhagen, Copenhagen, Denmark; 9 Department of Science and Environment, Roskilde University, Roskilde, Denmark; 10 Nic Waals Institute, Lovisenberg Diaconal Hospital, Oslo, Norway; 11 PsychGen Centre for Genetic Epidemiology and Mental Health, Norwegian Institute of Public Health, Oslo, Norway; 12 Center for Bioinformatics, Department of Informatics, University of Oslo, Oslo, Norway; 13 Lundbeck Foundation GeoGenetics Centre, GLOBE Institute, University of Copenhagen, Copenhagen, Denmark; 14 Department of Clinical Medicine, University of Copenhagen, Copenhagen, Denmark; 15 Division of Mental Health and Substance Abuse, Diakonhjemmet Hospital, Oslo, Norway; 16 Department of Psychiatry, Østfold Hospital Trust, Graalum, Norway

**Keywords:** Depression, Biomarkers

## Abstract

Genetic risk could be informative for identifying individuals at risk of depression with severe outcomes. With the novel approach of combined health care registries and self-report measures related to depression, the authors aimed to identify the impact of polygenic risk scores (PRS) for major depression on self report measures and a diagnosis of depression across diagnostic thresholds. The study sample comprised participants from the Norwegian Mother, Father and Child Cohort Study with linked information of depression diagnoses during 2006–2022 from health registries. Linear and logistic models were used to estimate the associations between PRS for major depression and self-reported measures related to depression (Symptom Checklist- 5, Satisfaction With Life Scale, Rosenberg Self-Esteem Scale), and a diagnosis of depression. Analyses were performed in groups stratified by level of depression severity defined by registrations in primary and specialist health care. Among the 105 623 individuals included in the study, mean (SD) age was 33.9 (5.3) and 58.5% were female. The associations between PRS for major depression and Rosenberg Self-Esteem Scale were more prominent in individuals with a history of inpatient status compared to outpatient status (β = 0.095, cluster robust 95% CI 0.029–0.162; *P* = 0.005) and status of primary care (β = 0.098, cluster robust 95% CI 0.035–0.160; *P* = 0.002). PRS for major depression were associated with a diagnosis of depression in all groups of depression severity, with highest effect sizes for more severe types (history of inpatient status: OR = 1.85, cluster robust 95% CI 1.75–1.94; *P* < 0.01). The results provide new knowledge of how PRS for major depression vary with depression severity.

## Introduction

Major depressive disorder (MDD) is a common mental disorder worldwide, with an estimated lifetime prevalence of 17–30% [1]. The disorder exhibits significant heterogeneity, characterized by differing severities and diverse trajectories. There is considerable inter-individual variation in socioeconomic decline, excess mortality, and comorbidity among individuals with MDD [2]. Although many individuals experience no more than one depressive episode, persistent symptoms and recurrence of MDD is common and present in up to 77% of individuals with MDD [3].

Factors associated with severe outcomes in MDD such as long duration to treatment, comorbid psychiatric and physical disorders [4], have been identified. However, prediction of severe outcome at an individual level remains a major challenge. The use of genetic association studies could increase the understanding of severe outcomes. However, the heritability of MDD is shown to be dependent on the phenotype definition [5, 6], challenging the development of genetic risk prediction tools. Polygenic risk scores (PRS) are developed from known genetic risk variants and represent a cumulative genetic risk for illness [7]. The clinical use of PRS in psychiatry is not yet convincing, and lags behind other medical fields [8]. To move forward, studies with longitudinal data and clinical information of illness heterogeneity are called for [8].

With the use of large cohorts of depression cases, PRS for major depression has previously shown to be higher in cases with more severe type, e.g. in cases with early onset, more fulfilled symptom criteria, recurrent episodes and chronic illness of depression [9]. In a smaller and younger cohort with longitudinal design, PRS for major depression predicted the development of depression [10]. In addition, PRS for major depression were higher in depression cases that developed more severe symptoms or had an early-onset [10]. PRS for major depression have previously been found to be associated with depressive symptom clusters [11, 12], in particular in cases with lifetime history of MDD [12]. The associations between PRS for major depression and different symptom clusters are important to investigate, given the heterogeneity of the disorder and the common delay of diagnosis after first symptoms [13]. A potential differentiated association between PRS for major depression and symptom clusters across diagnostic thresholds could inform the potential of PRS for major depression in future development of risk models. However, the association with symptom clusters related to depression has not previously been investigated in groups stratified by level of depression severity from register-based clinical diagnoses, in large cohorts. A major advantage of this approach is the available clinical information of a broad set of depression cases, from milder to more severe types, with long-term follow up. The access to universal health care in Norway and a very high coverage of data from primary and secondary care, offers an unique possibility to investigate a comprehensive set of diagnostic thresholds. Also, the investigation of PRS for major depression and association with a diagnosis of depression, has not previously been investigated in depression cases from a broad set of healthcare settings.

To provide new insight into the relationship between PRS for major depression and depression severity, the research aims of the current study were two-fold. First, to identify the impact of PRS for major depression on self report measures related to depression across diagnostic thresholds of depression from different healthcare settings. We hypothesize that PRS for major depression associates stronger for self-report measures related to depression in cases that developed a severe form of MDD. Second, to show the association between PRS for major depression and a diagnosis of depression across diagnostic thresholds from different healthcare settings. As an ancillary aim, we investigate the putatively specific contribution of PRS for major depression to the first and second aim, and include PRS for bipolar disorder and schizophrenia in the analyses. To validate the specificity for PRS for psychiatric disorders, we include analyses with PRS for height as a somatic comparator.

## Subjects and methods

### Participants

Mothers and fathers from The Norwegian Mother, Father and Child Cohort study (MoBa) were included in this study (Supplementary Note 1) [14]. Individuals were enrolled during pregnancy in 1999–2008. History of clinical diagnoses of mental disorders was available from linked data to the National Patient Registry (NPR, ICD-10 based) in specialist health care and the Norwegian Control and Payment of Health Reimbursements Database (CPHR, ICPC-2-based) in primary care. The NPR covers specialist health care across Norway, both inpatient and outpatient care, and contract specialists Norwegian Patient Registry (NPR) (helsedata.no). In our study sample, ICD-10 codes from somatic health care, mental health care, rehabilitation facilities and interdisciplinary addiction care were available for the period 2008–2022. The CPHR covers primarily contacts in primary care Norwegian Control and Payment of Health Reimbursements Database (KUHR) (helsedata.no). In our study sample, ICPC-2 codes in CPHR were available for the period 2006–2022. The NPR and CPHR have a high degree of coverage, since specialists in mental health care and general physicians are obliged to report to the registries in Norway [15].

Participants from MoBa answered questionnaires of lifetime depression in week 15 of gestation, defined as having at least 3 depressive symptoms simultaneously, including the symptom “felt depressed, sad”. Among 116 514 individuals, 22538 (19,3%) answered positive on lifetime depression, of which 10 891 (9,3%) individuals had no diagnosis of depression in the registries. This could be due to subthreshold symptoms of depression, symptoms due to another somatic or mental disorder, or depression treated in specialist or primary health care before the start of NPR/CPHR. In case of depression treated previously in healthcare, information of symptom severity and level of health care use would be missing. To avoid ambiguous groups of depression severity, we excluded individuals that answered positive on lifetime depression in questionnaires but had no registration of depression in health registries. The questionnaire for lifetime depression is provided in Supplementary Table 1.

In total, 105 623 individuals with complete data were included in the analyses (Supplementary Fig. 1). In sub-sample analyses, we excluded individuals with a history of other severe mental disorders registered in the NPR, resulting in 104 040 included individuals. The following ICD-10 codes were available for exclusion; organic mental disorders (F00-F02, F04, F06), bipolar disorder (F30–F31) and psychotic disorder (F20–F29).

### Ethics approval and consent to participate

All methods were performed in accordance with the relevant guidelines and regulations for the current study. The current study was approved by The Regional Committees for Medical and Health Research Ethics REK 2016/1226. Informed consent was obtained from all participants.

### Polygenic Risk Scores (PRS)

All individuals passed a standardized pipeline for genotyping (Supplementary Note 1). The PRS were calculated based on genome wide association studies (GWAS) for major depression [16], bipolar disorder [17], schizophrenia [18] and height [19], using the automatic mode of LDpred2 in the R-package bigsnpr [20], implemented in a container-based pipeline GitHub - comorment/containers: CoMorMent-Containers. All PRS were standardized to mean 0 and unit variance before entering statistical analyses.

### Phenotype Measures

#### Groups of depression severity

A diagnosis of depression was defined as at least one registration of ICD-10 codes F32 (depressive episode) and/or F33 (recurring depressive disorder) in the NPR during 2008–2022 or ICPC-2 codes P03 (feeling depressed) and/or P76 (depressive disorder) in CPHR during 2006–2022. Groups with separate levels of depression severity were defined by diagnostic codes in the registries. A history of no depression was defined as no registration of F32/F33 or P03/P76 in CPHR. *Level of symptom severity* was defined by the ICD-10 code in NPR. Mild MDD was defined as at least one registration of F32.0/F33.0 but no registration of F32.1/F32.2/F32.3/F33.1/F33.2/F33.3. Moderate MDD was defined as at least one registration of F32.1/F33.1 but no registration of F32.2/F32.3/F33.2/F33.3. Severe MDD was defined as at least one registration of F32.2 (severe without psychotic features), F32.3 (severe with psychotic features), F33.2 (severe without psychotic features) or F33.3 (severe with psychotic features). *The level of health care use* was defined by registrations in NPR and CPHR. Depression in primary care was defined as at least one registration of ICPC-code P03 and/or P76 in the CPHR but no registration of ICD-10 codes F32 or F33 in the NPR. MDD in outpatient care was defined as at least one registration of ICD-code F32 or F33 in the NPR, but no MDD in inpatient care. MDD in inpatient care was defined as at least one registration of ICD-code F32 or F33 with more than 0 treatment days in the NPR. Registrations from any area of specialist health care, i.e.somatic health care, mental health care, rehabilitation facilities and interdisciplinary addiction care were used. For more details of depression subcodes in the NPR, see Supplementary Note 2.

#### Self-reported measures related to depression

Self-reported measures related to depression were based on mean scores of the five items from the (Hopkins) Symptoms Check List (SCL-5) [21], five items of The Satisfaction With Life Scale (SWLS) [22] and four items of the Rosenberg Self-Esteem Scale (RSES) [23]. The SCL-5 comprises questions of symptoms present the past two weeks, SWLS and RSES comprises measures present and not limited to a time frame. These questionnaires are widely used in research for measures of mental distress. The participants answered the questionnaires at the same timepoint as self-report of lifetime depression (week 15 of gestation). The self-report measures were selected due to their relations with core depressive symptoms such as depressed mood and pessimistic views of the future (SCL-5), pessimistic views of life condition (SWLS), and ideas of unworthiness (RSES). A detailed description of self-report measures related to depression is given in Supplementary Note 3 and Supplementary Table 2.

### Statistical analyses

We investigated the association between each PRS and a diagnosis of depression using logistic regression, and mean scores of SCL-5, SWLS and RSES using linear regression. All self-reported measures related to depression were standardized before inclusion in the model. Analyses were stratified on groups with separate level of symptom severity and groups with separate level of health care use. To statistically evaluate whether the associations between PRS for major depression and self-report measures differed between the groups with different level of depression severity (i.e. level of symptom severity and level of healthcare), we performed multivariable linear regression models for SCL-5, SWLS and RSES including interaction terms between PRS for major depression and the levels of depression severity. The interaction terms captured the contribution of PRS for major depression for each level of depression severity, to inform the level specific association. 95% confidence intervals were calculated using robust standard errors clustered at the family level to account for potential dependency between observations from the same family, and deviations from distributional assumptions. All models were adjusted for year of birth in quintiles, sex and the first 10 genome-wide principal components, and all models were repeated in the sub-sample where individuals with a history of other severe mental disorders were excluded. All statistical tests were two-sided. The statistical tests were considered to not be independent and correction for multiple testing was not performed [24].

## Results

**Fig. 1:**
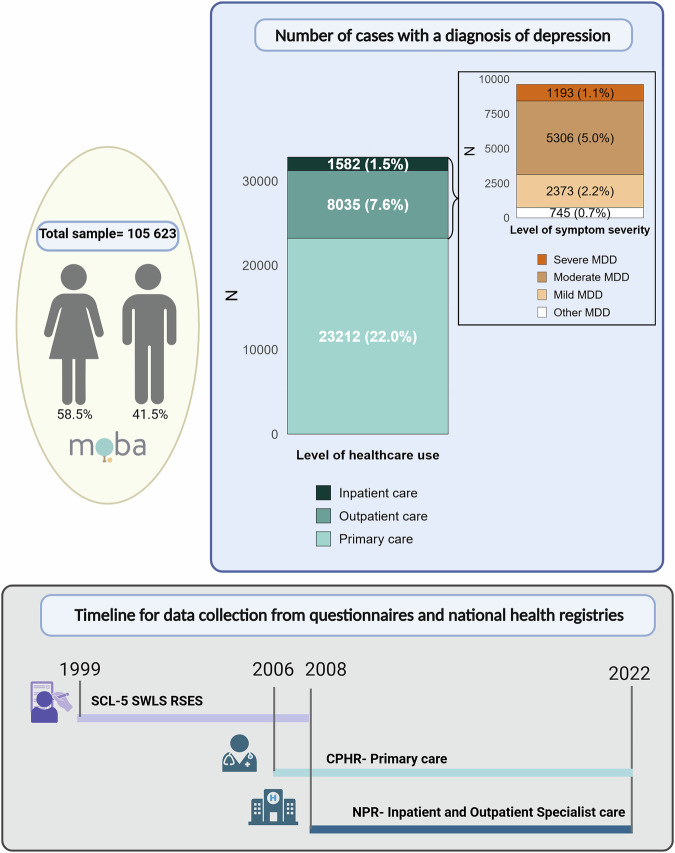
Overview of study participants. Overview of number of study participants, number of study participants with a diagnoses of depression in the registries and timeframe for data collection. Analyses were stratified on groups with separate level of health care use and with separate level of symptom severity. The definitions of groups are given in Table 1, in section Subject and Methods- Phenotype measures and in Supplementary Note 2. Moba- The Norwegian Mother, Father and Child Cohort study, SCL-5 The Selective items from the (Hopkins) Symptoms Checklist-5, SWLS The Satisfaction With Life Scale, RSES the Rosenberg Self-Esteem Scale, NPR National Patient registry, CPHR the Norwegian Control and Payment of Health Reimbursements Database. Created in BioRender. Haram, M. (2025) https://BioRender.com/m7yzxx6.

Among the 105 623 individuals with a mean age of 33.9 (5.3) at the start of follow-up in NPR, 58.5% were female and 31.1% had a registered diagnosis of depression. The number of individuals with depression of separate levels of severity are listed in Table 1 and shown in Fig. 1.

**Table 1:** Descriptive statistics of study participants.

	All			Females		Males	
	N			N		N	
	**Total sample**						
Total sample (%)	105623	100%		61807	58.5%	43816	41.5%
Age at start follow up in NPR, mean (SD)	33.9	5.3 SD		33.1	5.0 SD	35.1	5.5 SD
	**Total depression sample**^a^						
Any depression	32829	31.1%		23759	38.4%	9070	20.7%
Depression in primary care	23212	22.0%		16674	27.0%	6538	14.9%
MDD in specialist health care	9617	9.1%		7085	11.5%	2532	5.8%
	**Level of symptom severity**^b^						
Mild MDD	2373	2.2%		1794	2.9%	579	1.3%
Moderate MDD	5306	5.0%		3908	6.3%	1398	3.2%
Severe MDD	1193	1.1%		822	1.3%	371	0.8%
	**Level of health care use**^c^						
Depression in primary care	23212	22.0%		16674	27.0%	6538	14.9%
MDD in outpatient care	8035	7.6%		6022	9.7%	2013	4.6%
MDD in inpatient care	1582	1.5%		1063	1.7%	519	1.2%

*MDD* major depressive disorder, *SD* standard deviation, *NPR* National Patient Registry, *CPHR* Norwegian Control and Payment of Health Reimbursements Database.

^a^Any Depression- At least one registration of ICPC-2-code P03/P76 in the CPHR and/or at least one registration of ICD-10 codes F32/F33 in the NPR.

Depression in primary care- at least one registration of ICPC-code P03 and/or P76 in the CPHR, but no registration of MDD in the NPR.

MDD in specialist health care- At least one registration of ICD-10 codes F32/F33 in the NPR.This included the following subcodes: F32/F32.0/F32.1/ F32.2/F32.3/F32.8, /F32.9/F33/F33.0/F33.1/ /F33.2/F33.3/F33.4/F33.8/F33.9.

^**b**^Mild MDD- At least one registration of F32.0/F33.0 but no registration of moderate or severe MDD in the NPR.

Moderate MDD- At least one registration of F32.1/F33.1, but no registration of severe MDD in the NPR.

Severe MDD- At least one registration of F32.2/F32.3/F33.2/F33.3 in the NPR.

^**c**^MDD in outpatient care- at least one registration of ICD-code F32/F33 in the NPR, but no MDD in inpatient care.

MDD in inpatient care- at least one registration of ICD-code F32/F33 with inpatient contact F32/F33 in the NPR.

**Fig. 2:**
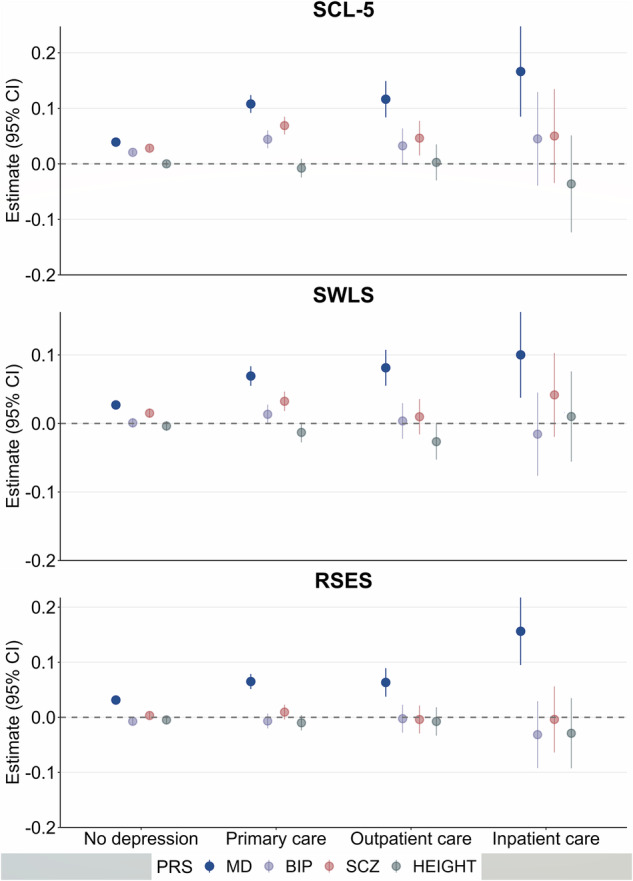
Associations between polygenic risk scores for major psychiatric disorders and self-reported measures related to depression, stratified by level of health care use. Associations between PRSs for major psychiatric disorders and height, and self-reported measures related to depression in stratified linear regressions. Estimates are given in standardized beta values with 95% cluster robust CI error bars. All models were adjusted for year of birth in quintiles, sex and the first 10 principal components. SCL-5 The Selective items from the (Hopkins) Symptoms Checklist-5, SWLS The Satisfaction With Life Scale, RSES the Rosenberg Self-Esteem Scale, PRS Polygenic Risk Score, MD Major Depression, BIP Bipolar Disorder, SCZ Schizophrenia, CI confidence interval.

**Fig. 3:**
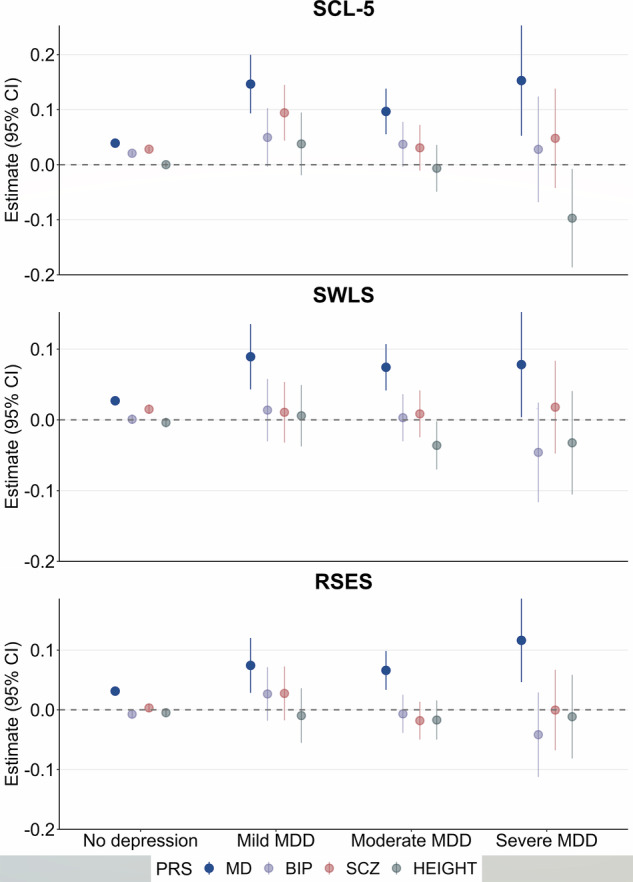
Associations between polygenic risk scores for major psychiatric disorders and self-reported measures related to depression, stratified by level of symptom severity. Associations between PRSs for major psychiatric disorders and height, and self-reported measures related to depression in stratified linear regressions. Estimates are given in standardized beta values with 95% cluster robust CI error bars. All models were adjusted for year of birth in quintiles, sex and the first 10 principal components. SCL-5 The Selective items from the (Hopkins) Symptoms Checklist-5, SWLS The Satisfaction With Life Scale, RSES the Rosenberg Self-Esteem Scale, MDD Major Depressive Disorder, PRS Polygenic Risk Score, MD Major Depression, BIP Bipolar Disorder, SCZ Schizophrenia, CI confidence interval.

**Fig. 4:**
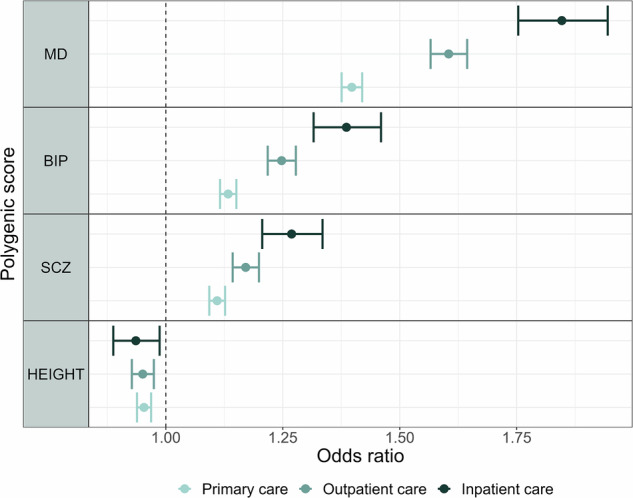
Associations between polygenic risk scores for major psychiatric disorders and a diagnosis of depression, stratified by level of health care use. Associations between polygenic risk scores for major psychiatric disorders and height, and a diagnosis of depression in stratified logistic regressions. Estimates are given in odds ratio with 95% cluster robust CI error bars. All models were adjusted for year of birth in quintiles, sex and the first 10 principal components. MD Major Depression, BIP Bipolar Disorder, SCZ Schizophrenia, CI confidence interval.

**Fig. 5:**
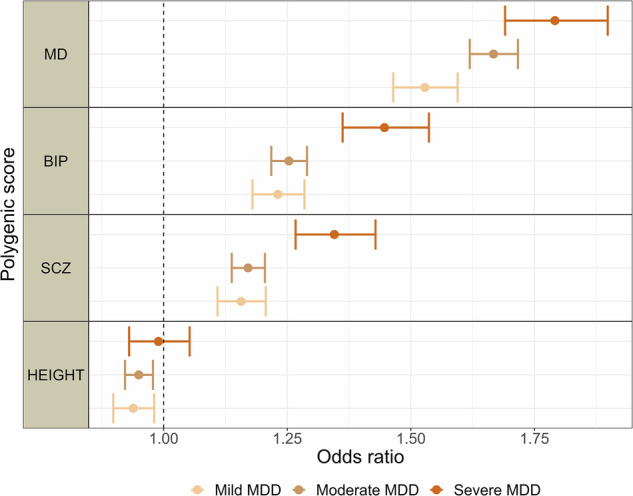
Associations between polygenic risk scores for major psychiatric disorders and a diagnosis of depression, stratified by level of symptom severity. Associations between polygenic risk scores for major psychiatric disorders and height, and a diagnosis of depression in stratified logistic regressions. Estimates are given in odds ratio with 95% cluster robust CI error bars. All models were adjusted for year of birth in quintiles, sex and the first 10 principal components. MDD Major Depressive Disorder, MD Major Depression, BIP Bipolar Disorder, SCZ Schizophrenia, CI confidence interval.

PRS for major depression, but not other PRS, were associated with higher levels of self-reported measures related to depression in all groups of depression severity (Fig. 2, Fig. 3, Supplementary Table 4). As initial findings did not show the same pattern between PRS for bipolar disorder or PRS for schizophrenia and self-report measures related to depression, the interaction analyses were performed in PRS for major depression only. They showed that PRS for major depression were associated with self-reported measures related to depression to a higher degree in most groups of depression severity levels compared to individuals with no history of depression (Fig. 2, Fig. 3, Supplementary Table 5). PRS for major depression were associated with RSES to a higher degree in individuals with experience of inpatient care compared to individuals with experience of outpatient care (β = 0.095, cluster robust 95% CI 0.029–0.162; *P* = 0.005) or primary care only (β = 0.098, cluster robust 95% CI 0.035–0.160; *P* = 0.002) (Fig. 2, Supplementary Table 5). PRS for major depression showed a gradient with depression severity in associations with a diagnosis of depression, with highest effect sizes in more severe cases (Primary care: OR = 1.40, cluster robust 95% CI 1.38–1.42; *P* < 0.01, Outpatient care: OR = 1.60, cluster robust 95% CI 1.57–1.64; *P* < 0.01, Inpatient care: OR = 1.85, cluster robust 95% CI 1.75–1.94; *P* < 0.01) (Fig. 4, Fig. 5, Supplementary Table 3). The same pattern was seen for other PRS except for height, but with lower effect sizes (Fig. 4, Fig. 5, Supplementary Table 3). We found negative associations between PRS for height and diagnoses of depression (Fig. 4, Fig. 5, Supplementary Table 3).

The majority of associations remained significant in the sub-sample excluding individuals with a history of severe mental disorders (Supplementary Fig. 2-5, Supplementary Table 3-5). The interaction between PRS for major depression and level of health care use in association with RSES did not reach level of statistical significance in the sub-sample (Supplementary Table 5).

## Discussion

In this large study with the novel design of combined self-report measures and long-term follow up in Norwegian health registries, we found new evidence of increased genetic contribution from PRS for major depression with increased depression severity across diagnostic thresholds. Higher polygenic risk for major depression was associated with higher ratings of distress, less satisfaction with life, and lower self-esteem. These associations were specific for PRS for major depression, as the same pattern was not seen for PRS for bipolar disorder and PRS for schizophrenia. We found stronger associations between PRS for major depression and lower self-esteem in depression cases with a history of inpatient care. The finding illustrates that individuals that developed more severe depression carry a greater genetic burden, that implicate their answers on self-report measures in a different time-frame than the registered depression. The stronger associations between PRS for major depression and lower self-esteem in depression cases with a history of inpatient care were not found in a sub-sample without individuals with severe mental disorders. This could indicate that PRS for major depression show strongest predictive potential in populations of individuals that will develop severe mental disorders. Further, PRS for major depression were associated with a diagnosis of depression with a gradient of depression severity, across a broad set of diagnostic thresholds. Also here, PRS for major depression showed stronger associations in contrast to PRS for bipolar disorder and PRS for schizophrenia.

### The predictive potential of PRS for major depression

While genetic association studies in depression are increasing discoveries of genetic risk variants, PRS for major depression have shown low predictive power and clinical utility [8]. Although the current study was not designed to investigate clinical use, our results indicate that PRS for major depression could associate stronger with self-report measures related to depression in individuals that will develop a severe form of depression. Given the low heritability of self-report symptoms in general [25], the results provide new knowledge on the potential applications of PRS for major depression. Since diagnostic information from the registries was not available in the same time-frame that self-report measures were provided, we were not able do investigate prediction per se. However, our proof-of-principle results show that further development of PRS for major depression for the current clinical use case is worth pursuing.

How PRS for depression should be calculated, either based on GWAS from a broad and heterogeneous set of cases, or a more homogenic group of cases, is debated [25]. A recent longitudinal investigation in youth found that PRS based on multitrait genetic loading showed stronger associations to depression trajectories, questioning the predictive value of PRS for major depression alone [26]. Further, PRS based on GWAS from a nonpsychotic treatment-resistant depression sample [27], has shown to be associated with treatment-resistance in a sample of psychotic depression cases [28]. Despite the many unresolved questions of how PRS for major depression should be calculated and used in a clinical setting, fine-grained knowledge of its predictive potential is evolving. In the GWAS meta-analysis used in the present study to calculate PRS for major depression, Als and colleagues found PRS for major depression to predict recurrence of first-episode depressive episodes [16]. Aligned with our findings, they found a weaker association for PRS for bipolar disorder, and no significant associations for other psychiatric PRS [16]. Zwicker and colleagues investigated the additional predictive value of PRS for major psychiatric disorders to family history of mood and psychotic disorders, on illness onset of the same disorders. They found PRS for major depression to predict illness onset in youths, but the prediction did not exceed known family history of illness. They found differences between separate PRSs, with PRS for neuroticism to provide additive predictive value to family history [29].

We found a positive interaction between separate levels of healthcare, but not levels of symptom severity, and self-reported measures related to depression. This contradictory finding could be due to health care organization in Norway. In accordance with Norwegian priority guidelines, individuals with mild depression do not have the right to receive specialist health care, but should receive treatment in primary care. Cases with a registered diagnosis of Mild MDD in our study are thus likely to receive treatment to an additional mental disorder, and not to represent solely milder cases of depression.

### Genetic heterogeneity of depression diagnoses

We validate previous findings of heterogenic polygenic liability to MDD based on subtypes [9]. Further, we provide new evidence to cover a broad set of diagnostic thresholds across different health care settings, showing increased genetic contribution from PRS for major depression with increased severity of the diagnosis. In a smaller study, Milaneschi etal. found MDD cases with typical symptoms (i.e., melancholic) to have overall stronger associations with PRS for major psychiatric disorders than MDD cases with atypical symptoms [30]. Furthermore, Power etal. found the explained variance by PRS for major psychiatric disorders to be greater for early-onset MDD compared to late-onset MDD [31]. The availability of register-data from both primary care and specialist health care with full coverage is unique for the Nordic countries, enabling the current investigation of PRS for major depression. In Norway, the general practitioner in primary care holds a gate-keeper role, and is often the first health care professional to be consulted. We found a high rate of depression, with 31.1% with a registered-based diagnosis during 16 years of follow-up. This is partly due to the high rate of depression registered in primary care, including more unspecific diagnoses of *feeling depressed*. Thus, our results also provide new insight into the prevalence of milder cases of depression, and how this group of cases associates with PRS for major depression. 1.5% of study participants were registered with a depression diagnosis in inpatient care and 7.6% in outpatient care, but not restricted to area of mental health care. ICD 10 codes registered in specialist health care during a hospitalization or outpatient contact in Norway must reflect the evaluation or treatment provided [32]. Thus, the registration of a depression code in other areas than mental health care reflects treatment seeking for other disorders, where evaluation and treatment of depression has reached focus during health care. In that manner, our sample ensures inclusion of a broad set of depression cases.

Although the use of PRS is still in a research phase, our results align with previous findings and add new knowledge to how PRS for major depression associates with depression severity. The current findings encourage further development of a multimodal risk model where PRS for major depression is included, to inform clinical decision making for treatment outcome in depression.

#### Strengths and Limitations

The availability of broad measures of symptom levels, and linkage to national health registries is a major advantage. Although the total length of follow-up was 16 years, the coverage period including all the registries was restricted to the years 2008–2022. Since individuals could have been registered with a diagnosis of depression in the health registries before this time period, stratification of diagnoses could be biased. To minimize this issue, we excluded individuals with ambiguous diagnostic status. This could have skewed our sample towards more severe cases, since individuals with chronic and recurrent symptoms are more likely to be captured by the registries during their life-course. If so, the sample would be prone to higher odds ratios in the association of polygenic risk scores of major depression and a diagnosis of severe depression, but also type II errors in the interaction analyses testing the association between PRS for major depression and self-report measures related to depression, in different categories of depression severity. Like many cohort studies, the MoBa study has some selection bias [14], and the participants are skewed towards higher socioeconomic status [33]. However, in comparison with nationwide register-based data from Sweden and Denmark, the prevalence of depression in MoBa has been found to be representative [2]. We found a negative associations between PRS for height and diagnoses of depression. Since there is a known genetic causal effect of height to socioeconomic position [34], PRS for height could potentially be skewed upwards in our sample. This could have attenatued the effect of PRS for height as a strictly somatic comparator.

## Conclusion

With the combined use of clinical information from long-term follow up in health registries, self-report measures and genetic data, we provide new knowledge of how PRS for major depression vary with depression severity. The results inform the potential of PRS for major depression for future development of risk models.

## Supplementary information

Supplementary Tables 1-5

Supplementary Information

## Data Availability

Data from the Norwegian Mother, Father and Child Cohort Study is managed by the Norwegian Institute of Public Health. Access requires approval from the Regional Committees for Medical and Health Research Ethics (REC), compliance with GDPR, and data owner approval. Participant consent does not allow individual-level data storage in repositories or journals. Researchers seeking access for replication must apply via www.helsedata.no.

## Code availability

The analyses code is available on request.
