# Supplementary material for: Associations of polygenic risk scores for major depression and depression severity: an investigation of 105 623 individuals with 16 years follow-up
Source: Mol Psychiatry. 2025 Sep 17;31(3):1325–32. doi: 10.1038/s41380-025-03243-2 (PMC12916310; doi:10.1038/s41380-025-03243-2)
Supplement: Supplementary file 2 — Supplementary Information [file 41380_2025_3243_MOESM2_ESM.docx]

**Supplemental Information**

**Supplementary Note 1** Description of The Norwegian Mother, Father and Child Cohort Study

**Supplementary Figure 1** Study sample

**Supplementary Note 2** Description of ICD-10 depression subcodes

**Supplementary Note 3** Description of self-report measures related to depression

**Supplementary Figure 2** Associations between polygenic risk scores for major psychiatric disorders and a diagnosis of depression, stratified on level of health care use, in a sub-sample without individuals with severe mental disorders.

**Supplementary Figure 3** Associations between polygenic risk scores for major psychiatric disorders and self-reported measures related to depression, stratified on level of health care use, in a sub-sample without individuals with severe mental disorders.

**Supplementary Figure 4** Associations between polygenic risk scores for major psychiatric disorders and a diagnosis of depression, stratified on level of symptom severity, in a sub- sample without individuals with severe mental disorders.

**Supplementary Figure 5** Associations between polygenic risk scores for major psychiatric disorders and self-reported measures related to depression, stratified on level of symptom severity, in a sub-sample without individuals with severe mental disorders.

**References**

**Supplementary Note 1**

**Description of The Norwegian Mother, Father and Child Cohort Study**

The Norwegian Mother, Father and Child Cohort Study (MoBa) is a population-based pregnancy cohort study conducted by the Norwegian Institute of Public Health. Participants were recruited from all over Norway from 1999-2008. The women consented to participation in 41% of the pregnancies. The cohort includes approximately 114.500 children, 95.200 mothers and 75.200 fathers. The current study is based on version 12 of the quality-assured data files released for research in 05.03.2024. The establishment of MoBa and initial data collection was based on a license from the Norwegian Data Protection Agency and approval from The Regional Committees for Medical and Health Research Ethics. The MoBa cohort is currently regulated by the Norwegian Health Registry Act.

Blood samples were obtained from both parents during pregnancy and from mothers and children (umbilical cord) at birth (1). Full details of the genotyping, pre-imputation quality control (QC), phasing, imputation, and post-imputation QC have been previously described (2). The final dataset passing post-imputation QC contains 6,981,748 autosomal SNPs and 207,569 individuals clustering with the 1000 Genomes European superpopulation. Included families have answered several questionnaires and are still continually asked to answer questions about illnesses and environmental factors related to health. The Medical Birth Registry (MBRN) is a national health registry containing information about all births in Norway. Linked data from the MBRN were used for year of birth information of the study participants.

**Supplementary Figure 1 Study sample**

**
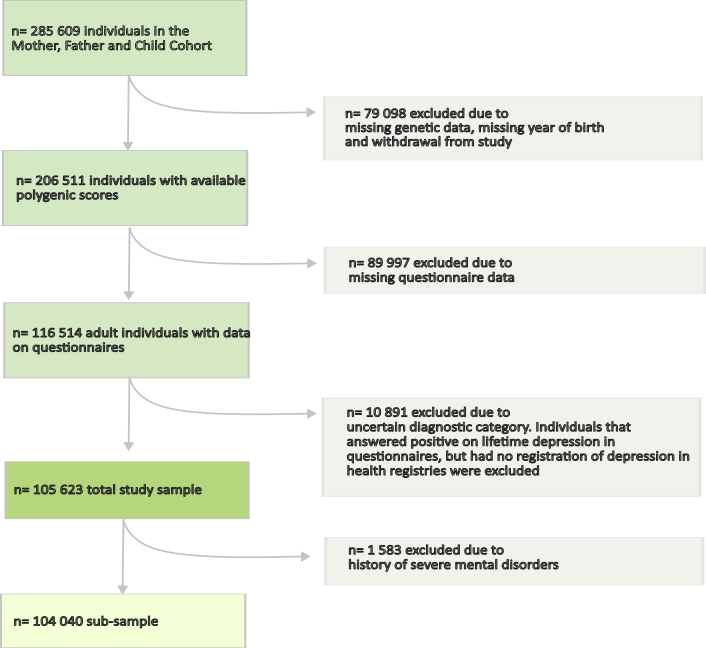
**

**Supplementary Note 2**

**Description of ICD-10 depression subcodes**

A diagnosis of depression in specialist health care was defined as at least one registration of ICD-10 codes F32/F33 in the NPR. This included the following subcodes: F32/F32.0/F32.1/ F32.2/F32.3/F32.8, /F32.9/F33/F33.0/F33.1/ /F33.2/F33.3/F33.4/F33.8/F33.9. Individuals with Mild/Moderat/Severe MDD and co-occurring registrations with other subcodes of MDD in specialist health care (F32/F32.8/F32.9/F33/F33.4/F33.8/F33.9) were included in the analyses of both “Level of symptom severity” and “Level of health care”. 745 individuals had no registration of Mild/Moderat/Severe MDD but other subcodes of depression only. These individuals were not included in analyses that investigated “Level of symptom severity”, but in analyses that investigated “Level of health care”.

**Supplementary Note 3**

**Description of self-report measures related to depression**

Participants in MoBa answered questionnaires in several time-points of each pregnancy. For the current study, mother and father questionnaires from week 15 of gestation were used <https://www.fhi.no/globalassets/dokumenterfiler/studier/den-norske-mor-far-og-barn--undersokelsenmoba/instrumentdokumentasjon/instrument-documentation-q1.pdf>, <https://www.fhi.no/globalassets/dokumenterfiler/studier/den-norske-mor-far-og-barn--undersokelsenmoba/instrumentdokumentasjon/instrument-documentation-q-father.pdf>

The Selective items from the (Hopkins) Symptoms Checklist-25 (SCL-25) comprises 25 questions scored on a 4-point Likert scale from “Not bothered” to “Very bothered”. In MoBa questionnaires, 5 items were selected from SCL-25 (SCL-5) for females and 8 items for males (SCL-8). For reasons of simplicity, we chose the same 5 items for males as were available for females in the current study. The 5-item selection has shown sufficient validity and reliability (3, 4). The Satisfaction With Life Scale (SWLS) comprises 5 questions scored on a 7-point Likert-scale from “Disagree Completely” to “Agree Completely”. The original version of the Rosenberg Self-Esteem Scale (RSES) comprises 10 questions on a 4-point Likert-scale from “Agree completely” to “Disagree completely”, and in MoBa Questionnaires four items were selected. Two of the questions are positively worded, while the other two questions are negatively worded. The four-item selection has shown sufficient reliability and validity (4). Before entering statistical analyzes, the sum-score of SWLS and the two negatively worded questions of RSES were inverted. Individuals that answered at least one question in the respective questionnaire were included. To minimize the impact of missing values, a mean score of for the respective questionnaire was used for further analyzes. All measures of SCL-5, SWLS and RSES were standardized before entering statistical analyses. In individuals with multiple pregnancies included in the cohort, a mean value of mean scores from the separate pregnancies was included in the analyzes.

**Supplementary Figure 2**

**Associations between polygenic risk scores for major psychiatric disorders and a diagnosis of depression, stratified by level of health care use, in a sub-sample without individuals with severe mental disorders**

**
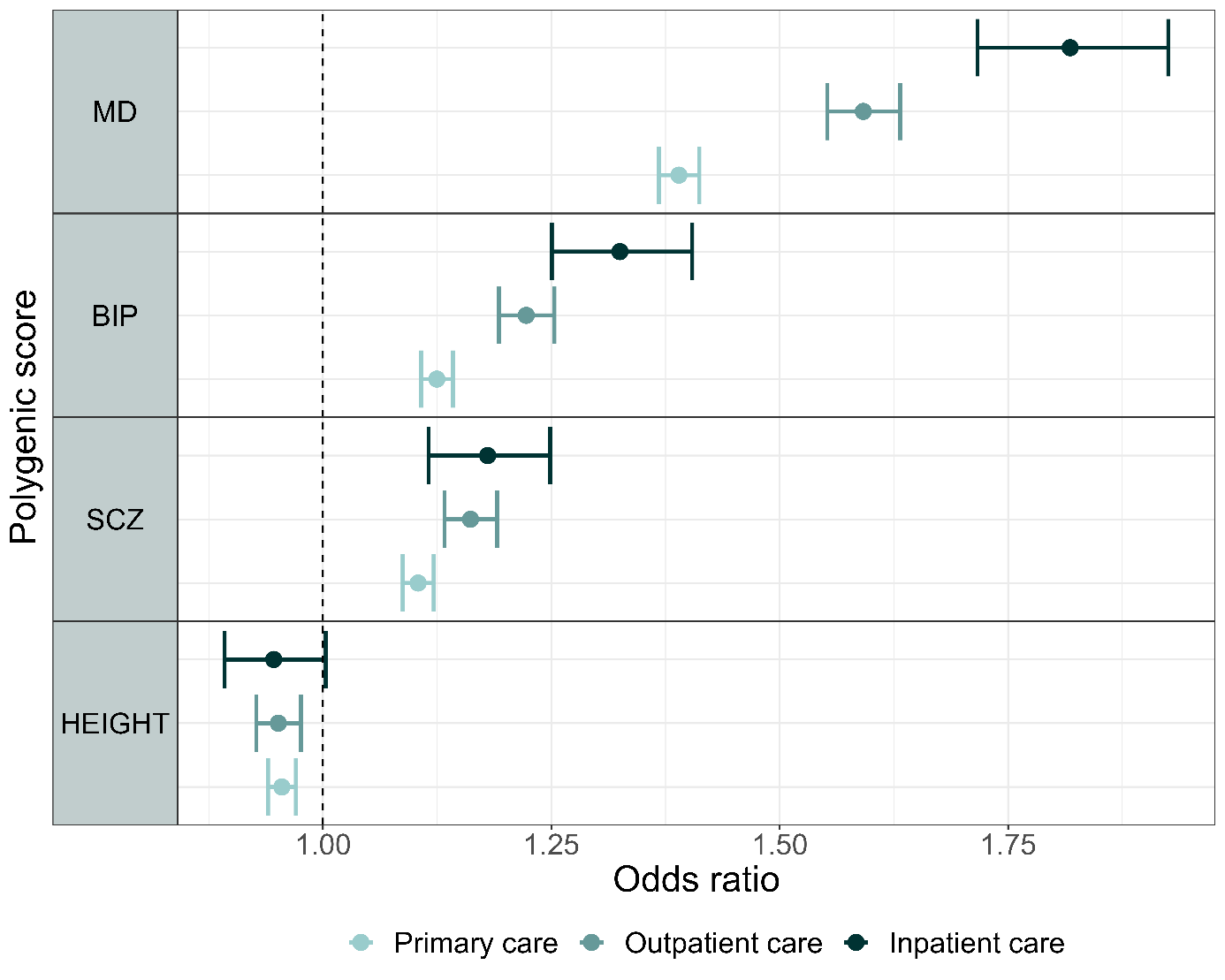
**

Associations between polygenic risk scores for major psychiatric disorders and height, and a diagnosis of depression in stratified logistic regressions. Estimates are given in Odds ratio with 95% cluster robust CI error bars. All models were adjusted for year of birth in quintiles, sex and the first 10 principal components.

MD- Major Depression, BIP- Bipolar Disorder, SCZ- Schizophrenia, CI- confidence interval.

**Supplementary Figure 3**

**Associations between polygenic risk scores for major psychiatric disorders and self-reported measures related to depression, stratified by level of health care use, in a sub-sample without individuals with severe mental disorders**

**
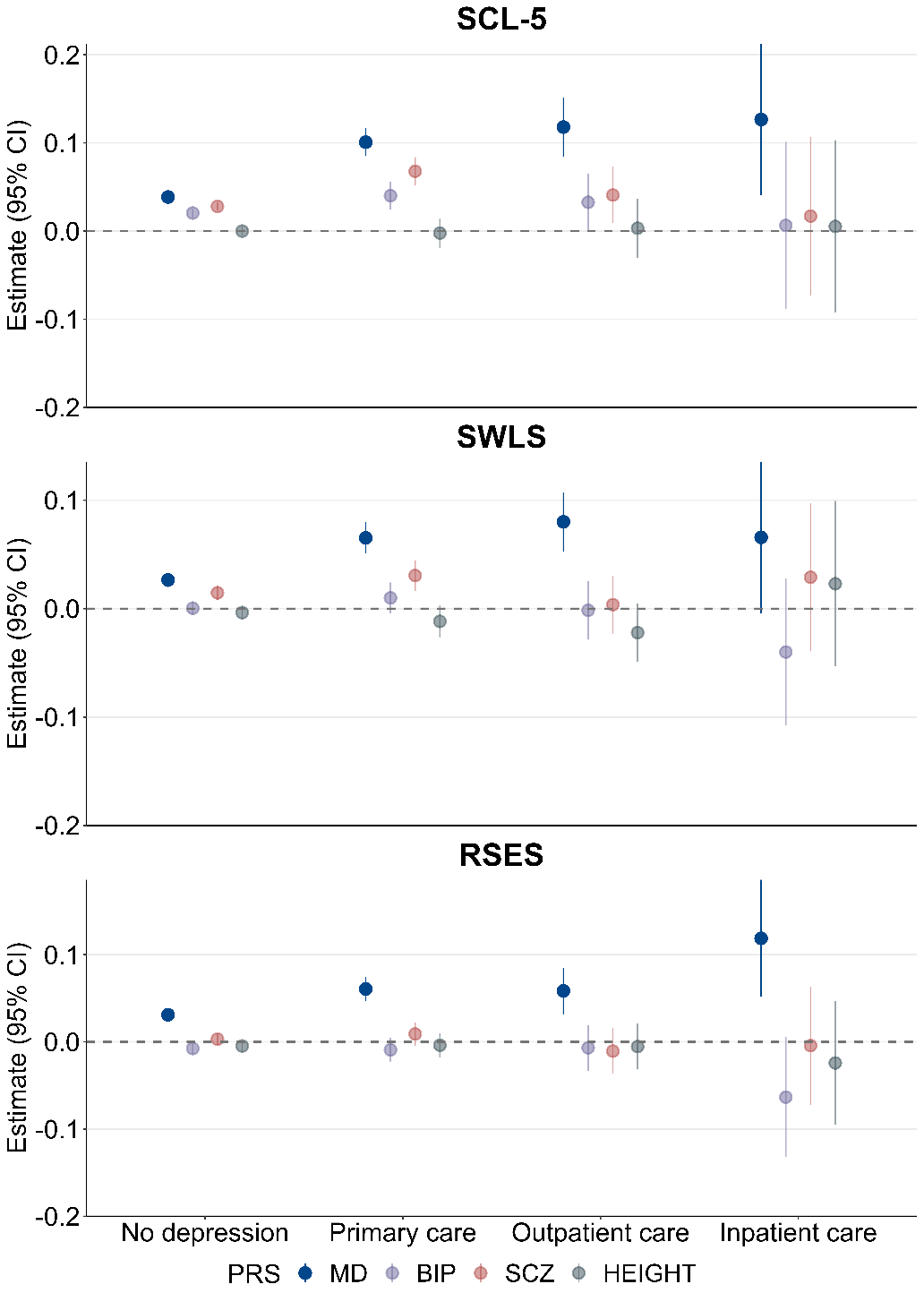
**

Associations between PRSs for major psychiatric disorders and height, and self-reported measures related to depression in stratified linear regressions. Estimates are given in standardized beta values with 95% cluster robust CI error bars. All models were adjusted for year of birth in quintiles, sex and the first 10 principal components.

SCL-5 -The Selective items from the (Hopkins) Symptoms Checklist-5, SWLS- The Satisfaction With Life Scale, RSES- the Rosenberg Self-Esteem Scale, PRS- Polygenic Risk Score, MD- Major Depression, BIP- Bipolar Disorder, SCZ- Schizophrenia, CI- confidence interval.

**Supplementary Figure 4**

**Associations between polygenic risk scores for major psychiatric disorders and a diagnosis of depression, stratified by level of symptom severity, in a sub- sample without individuals with severe mental disorders**

**
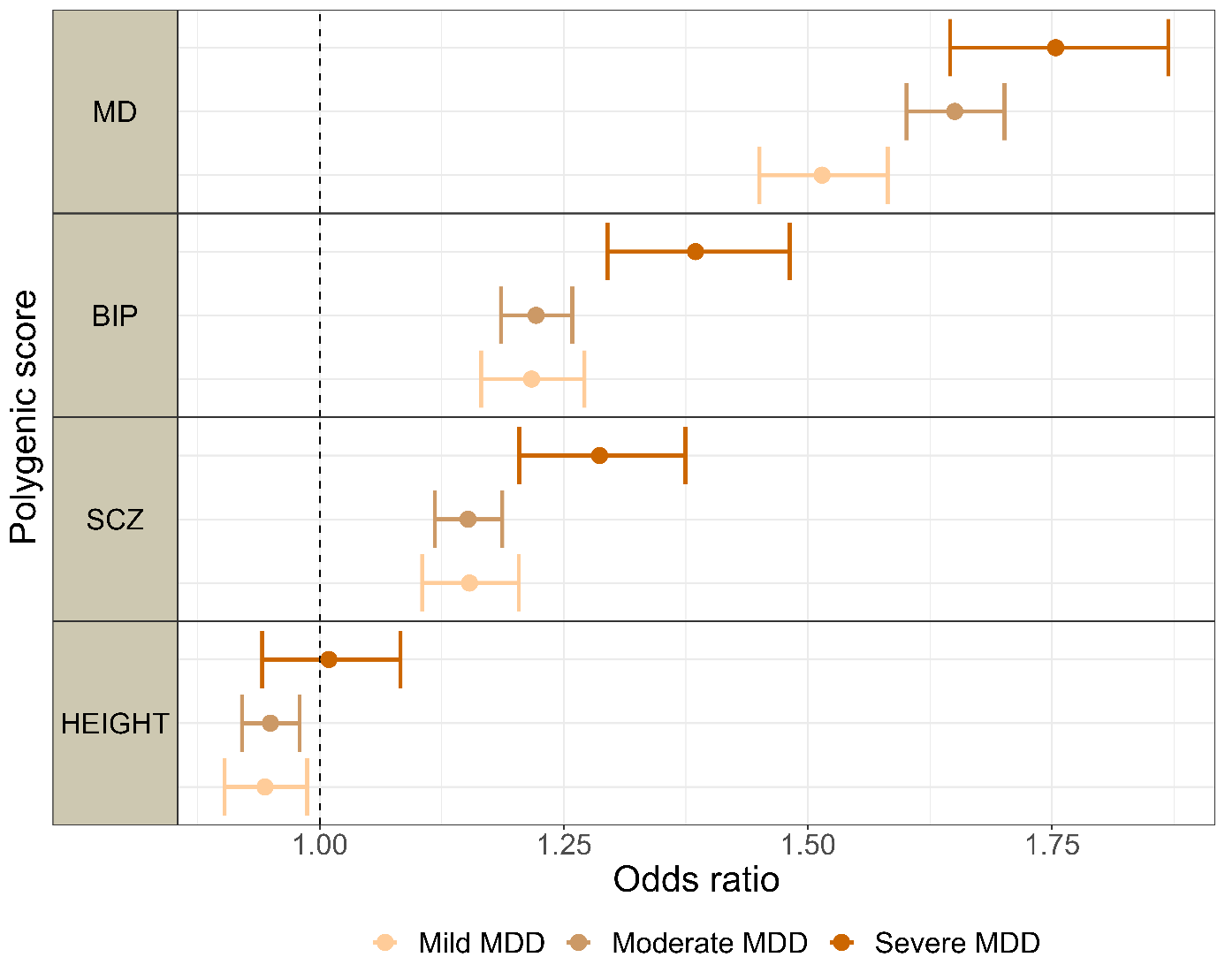
**

Associations between polygenic risk scores for major psychiatric disorders and height, and a diagnosis of depression in stratified logistic regressions. Estimates are given in odds ratio with 95% cluster robust CI error bars. All models were adjusted for year of birth in quintiles, sex and the first 10 principal components.

MDD- Major Depressive Disorder, MD- Major Depression, BIP- Bipolar Disorder, SCZ- Schizophrenia, CI- confidence interval.

**Supplementary Figure 5**

**Associations between polygenic risk scores for major psychiatric disorders and self-reported measures related to depression stratified by level of symptom severity, in a sub-sample without individuals with severe mental disorders**

**
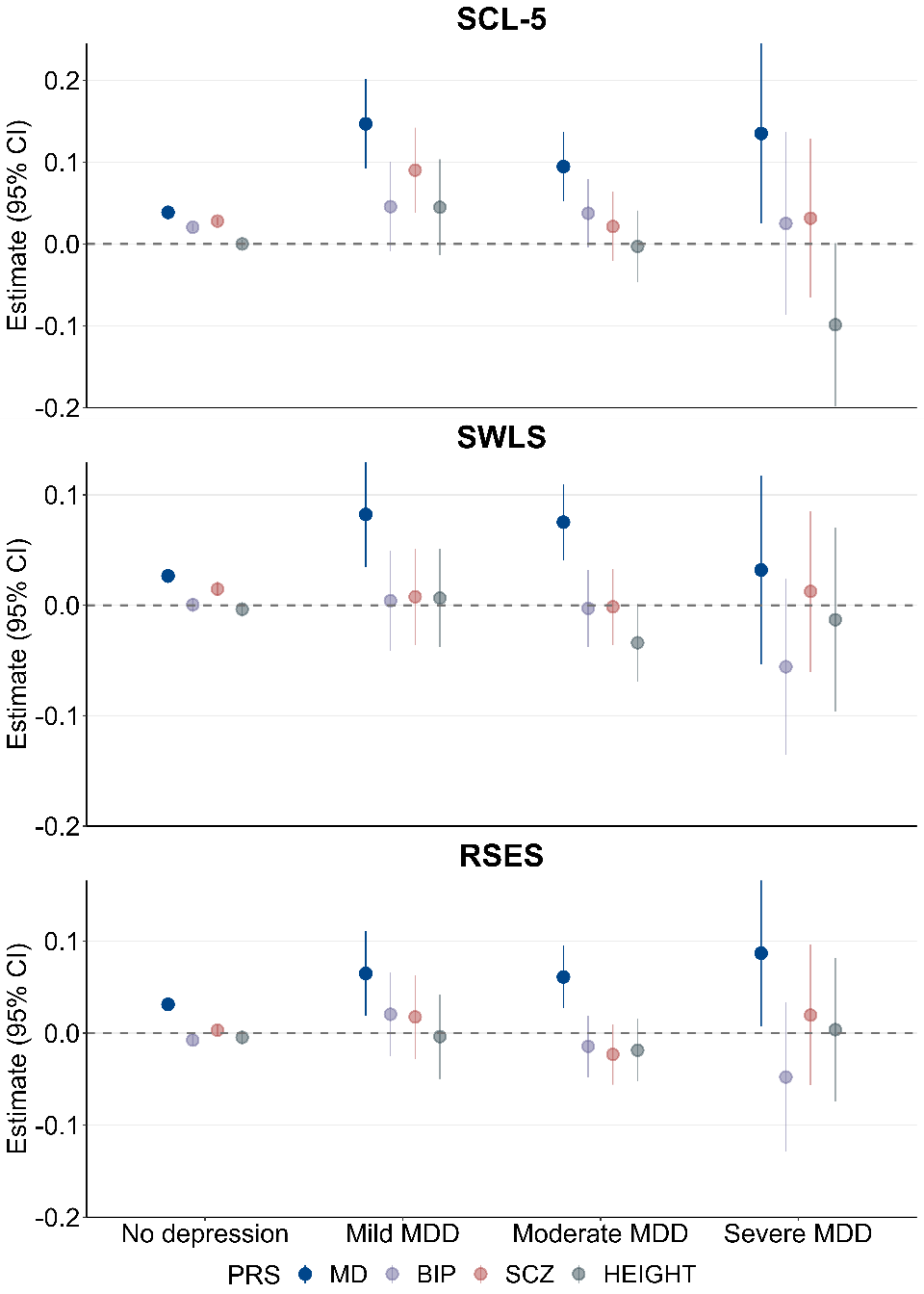
**

Associations between PRSs for major psychiatric disorders and height, and self-reported measures related to depression in stratified linear regressions. Estimates are given in standardized beta values with 95% cluster robust CI error bars. All models were adjusted for year of birth in quintiles, sex and the first 10 principal components.

SCL-5 -The Selective items from the (Hopkins) Symptoms Checklist-5, SWLS- The Satisfaction With Life Scale, RSES- the Rosenberg Self-Esteem Scale, MDD- Major Depressive Disorder, PRS- Polygenic Risk Score, MD- Major Depression, BIP- Bipolar Disorder, SCZ- Schizophrenia, CI- confidence interval.

**References**

1. Paltiel L, Anita H, Skjerden T, Harbak K, Bækken S, Nina Kristin S, et al. The biobank of the Norwegian Mother and Child Cohort Study – present status. Norsk Epidemiologi. 2014;24(1-2).

2. Elizabeth CC, Oleksandr F, Alexey AS, Zillur R, Aihua L, Lavinia A, et al. The Norwegian Mother, Father, and Child cohort study (MoBa) genotyping data resource: MoBaPsychGen pipeline v.1. bioRxiv. 2022:2022.06.23.496289.

3. Tambs K, Moum T. How well can a few questionnaire items indicate anxiety and depression? Acta Psychiatr Scand. 1993;87(5):364-7.

4. Tambs K, Røysamb E. Selection of questions to short-form versions of original psychometric instruments in MoBa. Norsk Epidemiologi. 2014;24(1-2).
